# Social Determinants of Health in Pediatric Asthma and Allergic Diseases: A Systematic Review

**DOI:** 10.3390/epidemiologia6030056

**Published:** 2025-09-11

**Authors:** Despoina Koumpagioti, Dafni Moriki, Barbara Boutopoulou, Pantelis Perdikaris, Konstantinos Douros

**Affiliations:** 1 Department of Nursing, University of West Attica, 12243 Athens, Greece; dkoumpagioti@uniwa.gr; 2 Respiratory and Allergy Unit, 3rd Pediatric Department, National and Kapodistrian University of Athens, General University Hospital “Attikon”, 12462 Athens, Greece; dmoriki@med.uoa.gr; 3 Department of Nursing, National and Kapodistrian University of Athens, 11527 Athens, Greece; bmpoutopoulou@gmail.com; 4 Department of Nursing, University of the Peloponnese, 22131 Tripoli, Greece; perdik@uop.gr

**Keywords:** environmental conditions, educational level, residence characteristics, neighborhood characteristics, geographic information system, asthma, allergic diseases, emergency department, hospitalization, children

## Abstract

Objectives: This study aimed to synthesize evidence of the influence of multilevel social determinants of health (SDOHs) on asthma and allergic disease outcomes and healthcare utilization in children and adolescents, with a specific focus on how these determinants generate or widen health inequities, through a systematic review of the current literature and evidence. Methods: A literature search was performed in the PubMed, EBSCO, and Scopus databases. The search period for all databases was from 1 January 2020 to 15 January 2025. Studies published in English that evaluated the association between at least one SDOH, as a primary exposure or effect modifier, and asthma and/or allergic disease outcomes and healthcare utilization in children and adolescents aged ≤ 18 years were included. A narrative synthesis was conducted to systematically explore and compare findings across studies, grouped by SDOH domains and disease outcomes. The grouping of SDOH domains was based on the framework established by the Healthy People 2030 Initiative. The selected studies underwent a quality assessment. Results: After the eligibility assessment, 44 studies were included in this review. Regarding study design, twenty-one studies were cohort, followed by eighteen cross-sectional, three ecological, and two case-crossover studies. Disease outcomes covered incidence, severity/exacerbations, lung function, and healthcare use in asthma, and analogous measures also reported for atopic dermatitis, allergic rhinitis, and food allergy. The most frequently studied domain of SDOHs was Neighborhood and Built Environment (n = 26), followed by Economic Stability (n = 24), Social and Community Context (n = 21), Healthcare Access and Quality (n = 12), and Education Access and Stability (n = 10). The vast majority of studies (n = 31) found positive associations between the examined SDOH factors and asthma and/or allergic disease outcomes and healthcare utilization. The most frequently evaluated SDOH with positive associations were neighborhood and residential conditions (n = 10), discrimination (n = 8), parental education (n = 7), housing quality (n = 6), air pollution (n = 6), and household income (n = 5). Risk-of-bias appraisal showed that the evidence base was largely at low risk, with most cohort, cross-sectional, ecological and case-crossover studies rated good quality, and only a few cohort studies classified as fair because of limitations in exposure assessment and residual confounding control. Conclusions: These findings highlight the urgent need for coordinated interventions and policies addressing social, environmental, and economic factors to reduce health disparities and improve outcomes for vulnerable children, while stressing the importance of interventional studies to provide stronger evidence.

## 1. Introduction

Social determinants of health (SDOHs), as defined by the World Health Organization (WHO), “are the non-medical factors that influence health outcomes” and include “the conditions in which people are born, grow, work, live, and age, and the wider set of forces and systems shaping the conditions of daily life” [1]. The Healthy People 2030 initiative categorized SDOHs into five domains: Economic Stability, Education Access and Quality, Health Care Access and Quality, Neighborhood and Built Environment, and Social and Community Context [2]. SDOHs have long been recognized as a key influence on a child’s growth, health, and development. They are most commonly observed in child populations as health inequities—unjust, unfair, avoidable, and unnecessary disparities that disproportionately affect vulnerable groups. These inequities arise from underlying social structures and economic, political, and legal systems, contributing significantly to child mortality and morbidity in both low- and high-income countries [3]. SDOHs play a crucial role in shaping overall well-being, affecting access to specialized healthcare, nutritious food, and safe, affordable housing that limits exposure to harmful environmental triggers. Extensive scientific research has consistently shown that individuals facing socioeconomic disadvantages—especially those living in poverty or belonging to underrepresented minority groups—experience considerably worse health outcomes related to allergic and immunologic diseases [4]. Recent evidence has increasingly linked SDOHs to variations in asthma prevalence, outcomes, and severity [5,6,7,8] and disparities in other allergic diseases [9,10].

A systematic review investigating the association between population-level social determinants of health (SDOHs) and pediatric asthma healthcare utilization found that most studies (n = 37) identified significant relationships between at least one SDOH and asthma healthcare utilization. Factors such as poverty, access to healthcare, access to primary care, enrollment in higher education, discrimination, environmental conditions, housing quality, and crime consistently showed significant associations [11]. Another recent systematic review examining the impact of SDOHs on pediatric asthma exacerbations showed that children with public or no health insurance, and those living in high-risk areas, had higher healthcare utilization due to asthma exacerbations [12]. Exposure to harmful environments, such as air pollution, has been associated with asthma-related hospitalizations [13], along with a higher risk of developing asthma or persistent wheezing [14]. Racial and ethnic disparities also persist, with Mexican American children being twice as likely to be hospitalized for asthma compared to White children—a rate comparable to that of Black children—reflecting the compounded effects of structural inequities embedded within the social determinants of health [15].

Allergic diseases among the pediatric population, such as atopic dermatitis (eczema), food allergy, allergic rhinitis, etc., have also been related to various SDOHs. A secondary cohort study analysis revealed that children from lower-income households were more likely to develop certain food allergies [9]. Additionally, African American children were more likely to live in rental housing, come from lower-income families, have caregivers with lower educational attainment, and be exposed to tobacco smoke—all of which were also linked to increased severity of atopic dermatitis (AD) [16].

A retrospective study of Florida Medicaid data (1997–2004) involving children under 18 years old showed that Hispanic children had 16% higher odds of being diagnosed with allergic rhinitis compared to White children, and 52% higher odds compared to Black children [17].

This study aimed to synthesize evidence on the influence of multilevel SDOHs on asthma and allergic disease outcomes and healthcare utilization in children and adolescents, with a specific focus on how these determinants generate or widen health inequities, through a systematic review of the current literature and evidence.

## 2. Materials and Methods

### 2.1. Design

This systematic review was conducted according to the Preferred Reporting Items for Systematic Reviews and Meta-Analyses (PRISMA) guidelines [18]. The present review was not registered.

The presentation of SDOHs was structured according to the Healthy People 2030 initiative’s categorization, which includes Economic Stability, Education Access and Quality, Health Care Access and Quality, Neighborhood and Built Environment, and Social and Community Context [2].

### 2.2. Literature Search

A literature search was performed in the PubMed, EBSCO, and Scopus databases. The search period for all databases was from 1 January 2020 to 15 January 2025. We limited our search to the past five years to capture the during and the post-COVID surge in SDOH research, extended our search to prior reviews that covered studies up to 2019, and ensured our synthesis reflected the most recent socio-economic context. Two reviewers (D.K. and D.M.) independently screened the studies based on the below eligibility criteria, resolving discrepancies through consensus. The search strategy is provided in Appendix A. Although terms directly related to healthcare access and quality were not explicitly included in the initial search strategy, relevant studies addressing these domains were identified during screening and included when they aligned with the broader inclusion criteria related to SDOHs.

### 2.3. Eligibility Criteria

The inclusion criteria for this systematic review encompassed observational (e.g., cohort, case-control, cross-sectional) and interventional studies; studies published in English from 1 January 2020 to 15 January 2025; and studies evaluating the association between at least one SDOH, as primary exposure or effect modifier, and asthma and/or allergic disease outcomes (e.g., prevalence, severity, exacerbations) and healthcare utilization (e.g., emergency department (ED) visits, hospitalizations) in children and adolescents ≤ 18 years.

We excluded studies that did not assess SDOHs concerning asthma and/or allergic diseases or had insufficient data; studies focusing exclusively on adults or mixed adult and pediatric populations; studies that included social variables exclusively as confounders without presenting their associations; and case reports, case series, reviews, systematic reviews, or meta-analyses.

### 2.4. Data Extraction

Two reviewers (D.K. and D.M.) independently extracted data from the selected studies, including study details (author, year of publication, country, and study design); characteristics of the study population (sample size and age); examined SDOHs; examined disease; disease outcomes and/or healthcare utilization; study findings on the relationship between SDOHs and disease outcomes and/or healthcare utilization; and covariates. Within this review, studies that treated race or ethnicity as exposure variables or covariates were interpreted as examinations of structural racism and categorized under the ‘Discrimination’ determinant within the Social and Community Context domain [11].

### 2.5. Data Analysis

Due to substantial heterogeneity in study designs, populations, exposure definitions, outcome measures, and statistical reporting formats, a meta-analysis was not feasible. The included studies varied widely in their methodologies and metrics, such as odds ratios, risk ratios, and narrative findings, which limited the possibility of meaningful statistical pooling. Therefore, a narrative synthesis was conducted to systematically explore and compare findings across studies, grouped by SDOH domains and disease outcomes.

### 2.6. Quality Assessment

Given the diversity of study designs, we applied design-specific tools to ensure a more appropriate assessment of risk of bias. Although a unified tool could improve consistency, it may not fully capture design-specific limitations. To assess the quality of the included cohort studies, we used the Newcastle–Ottawa Scale (NOS) tool [19]. The NOS is a widely accepted quality assessment tool that utilizes a “star system,” in which a study is evaluated based on three broad perspectives: the selection of the study groups, the comparability of the groups, and the ascertainment of the outcome of interest. Studies that received at least six stars were considered to be of good methodological quality [19]. For cross-sectional studies, we applied the AXIS tool (appraisal tool for cross-sectional studies), a 20-item checklist that addresses key areas such as study design, sample size justification, target population, sampling frame, sample selection, and measurement validity and reliability [20]. The 20 items of the AXIS tool were scored by assigning numerical values to categorical responses: “Yes” was scored as 1 and “No” as 0. Studies were classified as high quality if they achieved at least 70% of the total possible score (i.e., a minimum of 14 out of 20 points). Studies with scores between 60% and 69.9% were considered of fair quality, while those scoring below 60% were classified as low quality [20]. In ecological studies, there are no standard tools to assess study quality. Therefore, we used an adapted version of Dufault et al.’s 15-item checklist, which evaluates study design, statistical methods, and reporting, with a total score of 21 points (maximum 12 for design, 6 for statistics, and 3 for reporting) [21]. If data assumptions were not met, analytic methods received a score of 0; otherwise, they scored 1 [21]. For the assessment of case-crossover studies, we used a risk of bias 9-item scale based on the Cochrane Handbook and expert guidance, and adapted from the study by Ding et al. [22]. The criteria included 1. an appropriate crossover design, 2. a randomized treatment order, 3. the minimization of carry-over effects, 4. unbiased data, 5. allocation concealment, 6. blinding, 7. incomplete outcome data, 8. selective reporting, and 9. other biases [22]. Each item was rated as low risk (1 point), unclear, or high risk (0 points). The quality of all included studies in the systematic review was independently assessed by two reviewers (D.K. and D.M.).

## 3. Results

### 3.1. Study Selection

**Figure 1:**
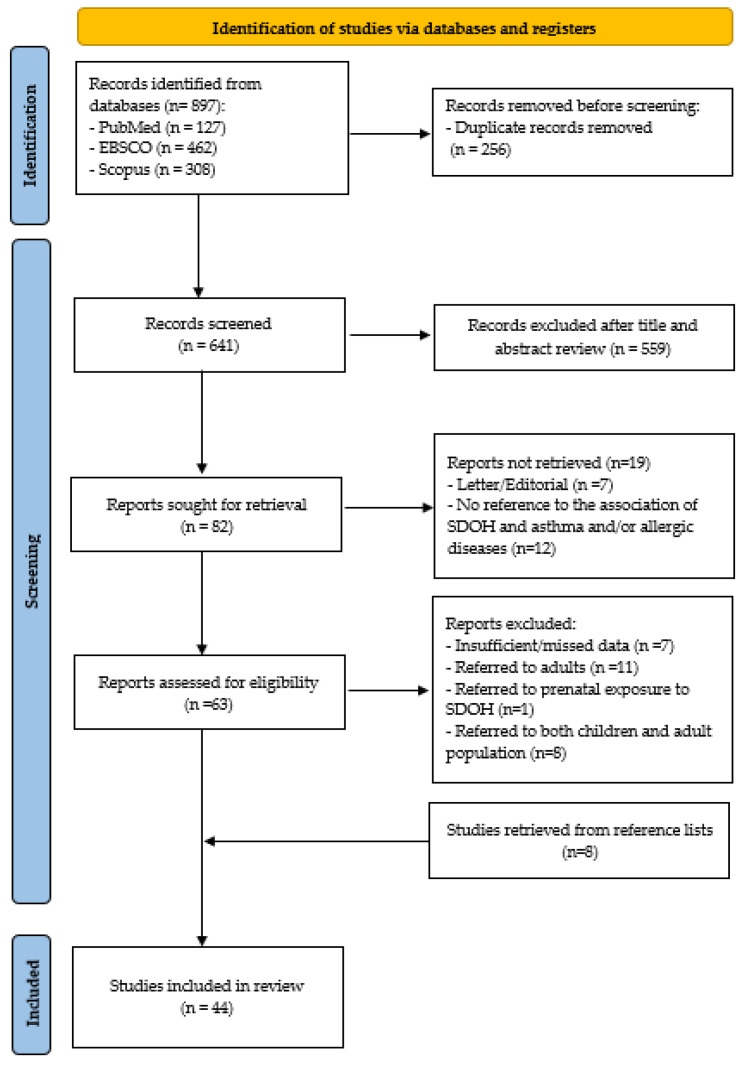
PRISMA diagram for the study selection process.

Initially, 897 records were identified from the database search. Before screening, 256 duplicate records were removed. The remaining 641 records underwent title and abstract screening, leading to the exclusion of 559 studies. Of the 82 reports sought for retrieval, 19 could not be retrieved. After further assessment, 63 reports were evaluated for eligibility, with 27 excluded. Additionally, eight studies were identified from reference lists. Ultimately, 44 studies were included in the systematic review. Figure 1 depicts the PRISMA diagram for the study selection process.

### 3.2. Study Characteristics

A total of 44 studies examined the SDOHs influencing asthma and allergic diseases among children and adolescents, along with their association with disease outcomes or healthcare utilization. The characteristics of the selected studies are presented in Table 1.

Most studies were conducted in the USA (n = 32), with two studies in Canada [23,24], two in Nigeria [25,26], and one study each in Korea [27], Greece [28], Spain [29], Denmark [30], Sweden [31], Italy [32], and Portugal [33]. One multinational study included participants from the United Kingdom, the Netherlands, Sweden, Australia, the USA, and Canada [34]. Regarding study design, twenty-one studies were cohort, followed by eighteen cross-sectional, three ecological, and two case-crossover studies. Most studies investigated multiple SDOHs (n = 31), while the remaining focused on a single SDOH category (n = 13). Thirty-five studies focused on asthma, five on AD [24,26,35,36,37], one on both asthma and AD [27], one on both asthma and allergic rhinitis [32], another on asthma, hay fever (allergic rhinitis), food allergy, and respiratory allergy [38], and one study examined food allergy alone [39].

**Table 1:** Characteristics of the selected studies.

Author	Country	Study Design	Study Population	SDOHs Examined	Disease Examined	Disease Outcome or Healthcare Utilization	Outcomes	Covariates
Adams & Knuth [40]	USA	Ecological	42 neighborhoods, children 0–17 years	Discrimination, urban living conditions, socioeconomic status, indoor environment	Asthma	ED visits	Positive association between ED visits and the percentage of Black children (r(35): 0.41; *p* < 0.05) and negative correlation for White residents (r(35): −0.44; *p* < 0.01). Percentage of non-Hispanic Black residents and ambient air temperatures were significant predictors of the rate of ED visits, with F(3,38): 22.354; *p* < 0.001, adjusted R2 = 0.61. Neighborhoods with lowest median income, such as the Bronx and Harlem, reported the highest asthma ED rates, with Harlem’s ZIP code 10037 reaching 597.2 ED visits per 10,000 children.	Percent of green space, homes with air-conditioning, percent of homes with maintenance defects, race/ethnicity
Antonogeorgos et al. [28]	Greece	Cross-sectional	1934 adolescents, mean age: 12.7 years	Parental education, indoor exposure (dampness and/or mold)	Ever had asthma, current asthma	Ever had asthma symptoms, current asthma	Significant association between current exposure to indoor dampness and/or mold and ever had asthma symptoms (adjusted OR: 1.52; 95% Cl: 1.06–2.19; *p* < 0.001) and current asthma (adjusted OR: 1.66; 95% Cl: 1.1–2.51; *p* < 0.001). Higher parental education was associated with 50% lower odds of indoor dampness/mold exposure and asthma than primary or secondary education (adjusted OR: 1.55; 95% Cl: 1.04–2.32 and adjusted OR: 1.96; 95% Cl: 1.06–2.19, respectively).	Sex, BMI, parental atopic history, adolescent’s history of allergic rhinitis and eczema, parental smoking, pet ownership, having an older sibling, cooking with fuels
Antoñón et al. [29]	Spain	Cross-sectional	349 children and adolescents, 6–14 years	Socioeconomic inequality (ARPR), parental educational attainment, residential setting, exclusive management by primary care (vs. involvement of allergologist)	Asthma	Asthma control, ED visits	No association between ARPR tertile with asthma control (*p* = 0.092). Significant association between medium/high maternal and paternal education and lower risk of unscheduled/urgent visits (OR: 0.50; 95% Cl: 0.27–0.95; *p* = 0.034 and OR: 0.51; 95% Cl: 0.28–0.94; *p* = 0.030, respectively).	Age, sex, ARPR stratum (low, medium, high), urban vs. rural setting, maternal educational attainment, paternal educational attainment, presence of smoker in the household, exclusive management by primary care (vs. involvement of allergologist), history of on-demand/urgent visits in 2021
Aratani et al. [41]	USA	Cohort	47,657 children, 0–6 years	Discrimination	Asthma	ED visits	Among English-speaking families, Black individuals were less likely to be hospitalized during their first asthma ED visit (OR: 0.787; 95%CI: 0.715–0.866) but more likely to return to the ED (OR: 1.291; 95%CI: 1.205–1.383) vs. White individuals. English-speaking Asian/Pacific Islanders had a higher likelihood of hospitalization (OR: 2.150; 95%CI: 1.827–2.530) vs. White individuals. Among non-English speaking families, Hispanic and Asian/Pacific islanders were more likely to be hospitalized during their first asthma ED visit (OR: 1.427; 95% Cl: 1.332–1.529 and OR: 1.605; 95% Cl: 1.213–2.124, respectively), non–English-speaking groups: less likely to return to the ED vs. English-speaking White individuals.	Age, sex, race/ethnicity, air basin region, disposition status at first visit, family language
Aris et al. [42]	USA	Cohort	10,516 children, median age at follow-up: 9.1 years	COI, SVI	Asthma	Asthma incidence	High and very high COI in early life vs. very low COI associated with lower asthma incidence (adjusted IRR: 0.87; 95%CI: 0.75–1.00). No association between SVI and asthma incidence.	Sex, race/ethnicity, birth year, BMI in early childhood, maternal educational level, annual household income during pregnancy, maternal prepregnancy BMI, prenatal cigarette smoking, prenatal secondhand smoke exposure, parental history of asthma, parity, mode of delivery, gestational age, rurality of residence
Aryee et al. [43]	USA	Cross-sectional	8653 children, 0–17 years	Neighborhood support, safety, resources, and quality	Asthma	Asthma prevalence, asthma severity	Children living in neighborhoods with high support, safety, and quality had less asthma prevalence (OR: 0.9; 95% Cl: 0.8–1.0; *p* = 0.02, OR: 0.7; 95% Cl: 0.5–0.9; *p* = 0.02, OR: 0.9; 95% Cl: 0.8–1.0; *p* = 0.03, respectively). No associations between neighborhood scores and asthma severity.	Age group, sex, race/ethnicity, family income level
Baek et al. [44]	USA	Retrospective cohort	902 children and adolescents, 5–18 years	Air pollution (PM_2.5_, O_3_), SVI	Asthma	Hospitalizations	Significant association between elevated average O_3_ levels in children’s residential neighborhoods with increased asthma-related hospitalizations (OR: 1.78; 95% Cl: 1.01–3.14; *p* = 0.045).	Age, gender, ethnicity, type of insurance, medication use, length of stay in hospital, season of admission, year of admission
Caffrey Osvald et al. [31]	Sweden	Cohort	88,540 children, mean asthma/wheeze onset age: 2.4 years	Parental education, parental income	Current asthma	Incidence of asthma	Weak association between lowest maternal education and current asthma at 5 years vs. highest education (adjusted OR: 1.05; 95% Cl: 1.00–1.11). No association between the lowest maternal income and current asthma at 5 years vs. the highest maternal income (adjusted OR: 0.98; 95% CI: 0.94–1.02).	Sex, parity, maternal country of birth, parental age at child’s birth, preterm birth, small for gestational age, number of siblings born during the first 5 years, maternal smoking during pregnancy
Choragudi et al. [37]	USA	Cross-sectional	149,379 children, 0–17 years	Discrimination	Eczema	Healthcare utilization trends	White children with eczema had a higher annual increase in well-child checkups and an upward trend in seeing a medical specialist, unlike other minority groups with stagnant trends.	Age, gender, race/ethnicity
Commodore et al. [45]	USA	Cohort	855 children, mean age: 6.9 years	Neighborhood traffic, neighborhood characteristics, home environment amenities	Asthma	Asthma symptoms, asthma-like symptoms	Children with high neighborhood traffic density had higher odds of having asthma/asthma-like symptoms vs. children without (adjusted OR: 2.1; 95% Cl: 1.12–3.62).	Age, sex, race/ethnicity, maternal education, family history of asthma, obesity, exposure to secondhand smoke, household pets, prescribed asthma medication, presence of public park, presence of play equipment at home, respiratory allergy diagnosis, gestational age at delivery, urban vs. rural census tract
Correa-Agudelo et al. [46]	USA	Retrospective cohort	31,114 children and adolescents < 18 years	Environmental-level and individual factors	Asthma	ED visits	7% increase in ED visits due to Medicaid insurance vs. commercial (1.07; 95% Cl: 1.03–1.1). Significant association between PM_2.5_ level, pollen, and outdoor mold exposure and increased rate of asthma ED visits for both European American and African American children (*p* < 0.001). No association between race and asthma ED visits (sβ: 0.006; *p* = 0.796).	Age at ED visit, gender, race, insurance type, neighborhood socioeconomic deprivation, proximity to hospital, proximity to major roads, proportion of greenspace, PM_2.5_ levels, pollen exposure, outdoor mold exposure
Faison et al. [47]	USA	Cohort	632 children, median age: 7 years	Housing insecurity (recent address changes)	Asthma	ED visits	No association between housing insecurity (recent address change) and asthma-specific 30-day or 90-day revisits (*p* = 0.114), in multivariate analysis.	Age, sex, race/ethnicity, insurance status, asthma severity, number of outpatient and inpatient encounters during the past 12 months
Grant et al. [48]	USA	Cohort	155 children, 5–17 years	Indoor allergen and pollutant exposures (airborne mouse allergen, bedroom floor mouse allergen, cockroach, dog, cat, nicotine, PM_2.5_, PM_2.5–10_)	Persistent asthma	Air trapping, airflow limitation	Association of airborne and bedroom floor mouse allergen concentrations with air trapping, but not with airflow limitation (OR:1.19; 95% Cl:1.02–1.37; *p* = 0.02 for each 2-fold increase in airborne mouse allergen; OR: 1.23; 95% Cl: 1.07–1.41; *p* = 0.003 for each 2-fold increase in bedroom floor mouse allergen). No association between exposures to cockroach (*p* = 0.55), dog (*p* = 0.37), cat (*p* = 0.66), PM_2.5_ (*p* = 0.73), PM_2.5–10_ (*p* = 0.55), and nicotine (*p* = 0.81) and air trapping or airflow limitation.	Time (0, 3, 6 months), treatment group, time × group, (age, sex, race, household income, insurance type, medication adherence: no material confounding)
Grunwell et al. [49]	USA	Cohort	1403 children, 6–17 years	Neighborhood hot spots, COI, SVI	Life-threatening asthma	PICU admissions, hospital length of stay	Children with critical asthma in PICU hot spots had higher SVI (0.67 vs. 0.46) and lower COI (17 vs. 48), with more inpatient bed days (14.8 vs. 8.8) and a higher bed day rate per 1000 (13.0 vs. 5.0; *p* < 0.0001).	Age, sex, race/ethnicity, insurance status, primary language, insurance type, hospital campus, prior asthma history, medical complexity status, acute-care interventions
Hauptman et al. [50]	USA	Cohort	350 children, mean age: 7.9 years	Major roadway proximity	Asthma	Asthma symptom days, health care utilization, asthma control	Significant association between major roadway proximity and increased asthma symptom days (*p* < 0.01). Beyond a distance of 100 m from a major roadway, there was a 29% lower likelihood of experiencing a symptom day in the past two weeks for every additional 100 m increase in distance (OR 0.71; 95% CI: 0.58–0.87; *p* < 0.01). Children living farther from major roadways had significantly lower healthcare utilization (OR 0.63; 95% CI: 0.47–0.85; *p* < 0.01) and were less likely to have poor asthma control (OR 0.80; 95% CI: 0.69–0.94; *p* < 0.01).	Age, sex, race/ethnicity, annual household income, use of asthma controller medication at baseline, environmental tobacco smoke exposure, upper respiratory infection in the past 2 weeks, seasonality
Huang et al. [51]	USA	Case-crossover	54,632 children and adolescents ≤ 18 years	Ambient air pollution (PM_2.5_, O_3_)	Asthma	Asthma exacerbations	Association between higher air pollution with more asthma exacerbation—PM_2.5_ during both warm and cold months (OR: 1.05; 95% Cl: 1.02–1.07 and OR: 1.03; 95% Cl: 0.98–1.08, respectively) and O_3_ during cold months (OR: 1.08; 95% Cl: 1.02–1.14).	Age, gender, race/ethnicity, public payer source for clinical visit, clinical setting (outpatient, ED, inpatient), comorbid allergic conditions, temperature, relative humidity, wind speed, precipitation, aeroallergen concentrations
Joy et al. [25]	Nigeria	Cross-sectional	66 children, mean age: 11.6 years	SES, mother’s education and employment status, number of children in the household	Asthma	Asthma control	No association between SES (*p* = 0.95), mother’s education (*p* = 0.76), mother’s employment status (*p* = 0.307), household number (*p* = 0.77), and asthma control.	-
Jung et al. [52]	USA	Cohort	240 children, mean age: 10.9 years	Home and school pollutants (PM_2.5_, NO_2_)	Moderate-to-severe asthma	Asthma severity, lung function	No association between home and school exposure to PM_2.5_ and asthma severity. Among children in redlined neighborhoods, higher levels of PM_2.5_ were associated with more severe asthma (*p* < 0.005). No significant association between home and school pollutants and lung function or asthma severity in children living in non-redlined neighborhoods (*p* > 0.005) *.	Age, sex, race/ethnicity, study site location, randomization status (mepolizumab vs. placebo), environmental tobacco smoke exposure, season, height (for lung function outcomes), proximity to highways, O_3_ levels, number of positive skin tests to indoor aeroallergens, ICS plus LABA use
Khan et al. [53]	USA	Cross-sectional	1959 children, mean age: 9.2 years	Cluster membership reflecting combined housing and neighborhood characteristics	Asthma	Asthma exacerbation	Children residing in high-density rental areas were 2.33 times more likely to experience asthma exacerbations, vs. those in newer, lower-density areas (adjusted OR: 2.33; 95% CI: 1.25–4.44).	Age, sex, race/ethnicity, household poverty level, household composition
Kim et al. [54]	USA	Retrospective cohort	69,118 children, 0–18 years	Discrimination, neighborhood SES	Asthma	ED visits, hospitalizations, PCP	African American children had higher ratio of asthma ED visits to outpatient visits (OR: 1.32; 95%CI: 1.08–1.62) and asthma hospitalizations to outpatient visits (OR: 1.50; 95%CI: 1.30–1.73), higher ratio of ED visits (OR: 1.36, 95%CI: 1.10–1.68) and hospitalizations (OR: 1.47; 95%CI: 1.26–1.71) vs. PCP visits.	Age, sex, race/ethnicity, Asthma Medication Ratio, neighborhood-level variables, severity of illness, spoken language
Kim et al. [27]	Korea	Cross-sectional	2070 children with asthma < 12 years, 980 with AD > 13 years	SES (Q1: lowest household income, Q2: middle, Q3: high, Q4: highest)	Allergic asthma, atopic dermatitis	Healthcare utilization	Significant association between highest household income group Q4: highest) with higher healthcare utilization for allergic asthma (OR: 1.36; 95% CI: 1.08–1.71; *p* < 0.001) and AD (OR: 1.58; 95% CI: 1.41–1.76; *p* < 0.001) vs. other income groups (Q1–Q3).	Age, gender, parental education, parental employment, insurance type, residential location
Le et al. [38]	USA	Cross-sectional	5042 children, 0–17 years	Discrimination, income, parental education, healthcare access, food security/insecurity	Ever had asthma, current asthma, hay fever, food allergy, skin allergy, respiratory allergy	Diseases’ prevalence	Significant associations between financial instability and food allergy prevalence in Asian Indian children (*p* = 0.02), lower education level with hay fever and respiratory allergy for non-Hispanic “other Asian” American children (*p* = 0.001 and *p* = 0.006, respectively), rent/other housing arrangements and skin allergy for non-Hispanic Filipino children (*p* = 0.01). Number of unfavorable SDOHs were lowest among non-Hispanic Asian Indian and Chinese children (mean: 0.7) and highest among non-Hispanic “other Asian” American children (mean: 1.2). Association between inaccessible healthcare and a greater likelihood of skin allergies in Chinese children, hay fever in Asian Indian children, and any allergic disease in both subgroups.	Age, sex, ethnicity subgroup, nativity, parental nativity, survey year
Mahdavinia et al. [39]	USA	Prospective cohort	239 AA children, 425 Whites, 0–12 years	Discrimination	Asthma, allergic rhinitis, eczema, food allergy	-	Higher odds of allergy to finfish (adjusted OR: 2.54; *p* < 0.01), and shellfish (adjusted OR: 3.10; *p* < 0.001) in AA children vs. Whites. Higher adjusted odds of asthma than Whites (asthma prevalence of 60.5% in AAs and 27.2% in Whites, OR: 2.70, *p* < 0.001).	Age, gender, race/ethnicity, annual income, study site
Mersha et al. [55]	USA	Prospective cohort	695 children, 1–16 years	Socioeconomic hardship, indoor and outdoor environmental exposure	Asthma	Asthma readmissions	Significant association between greater family hardship and asthma readmissions. (sβ: 0.013; *p* = 0.02 and sβ: 0.26; *p* < 0.001, respectively). No association between African ancestry and asthma readmissions, after adjusting for mediators (sβ: 0.035; *p* = 0.388).	Age, sex
Molina et al. [56]	USA	Cross-sectional	664 children, 2–12+ years	Residential instability, neighborhood deprivation	Asthma	ED readmissions, severe hospitalizations, re-hospitalizations	Significant association between increasing residential instability and worse asthma outcomes (more severe asthma (OR: 1.18; 95% Cl: 1.05–1.32; *p* = 0.004); higher risk of 365-day ED readmission (HR: 1.10; 95% Cl: 1.05–1.15; *p* < 0.001); and higher risk of 365-day re-hospitalization (HR: 1.09; 95% Cl: 1.03–1.14; *p* = 0.002). No association between ADI and asthma readmissions (*p* > 0.05).	Age, sex, race, insurance type
Pollack et al. [57]	USA	Prospective cohort	123 children, median age: 8.4 years	Neighborhood poverty level, housing mobility	Asthma	Asthma exacerbations, asthma symptoms	Children living in high-poverty neighborhoods experienced at least one exacerbation per three-month period. Exacerbation rate dropped to 8.5% (adjusted difference: −6.8%; 95% CI: −11.9% to −1.7%; *p* = 0.009), after relocating to low-poverty areas and the number of symptom days decreased from 5.1 days in the past week to 2.7 days (adjusted difference: −2.37 days; 95% CI: −3.14 to −1.59; *p* < 0.001).	Age, sex, race/ethnicity, asthma medication use, allergen sensitization, indoor exposures, rental assistance status before enrollment, seasonality of asthma symptoms
Reimer-Taschenbrecker et al. [36]	USA	Cross-sectional	216 children, 5–17 years	Insurance type, family income, discrimination, parental education, Deprivation Index (DI)	Atopic dermatitis	Severity, patient-reported outcome	No association between DI and AD severity, and income, parental education, and discrimination with the odds of moderate-to-severe AD vs. mild AD.	Age, sex, residential setting, smoke exposure, atopic comorbidities, breastfeeding history, pet exposure
Rennie et al. [23]	Canada	Cross-sectional	280 children, mean age: 10.9 years	Domestic risk factors (damp housing conditions, household heating, passive smoking exposure)	Atopic, non-atopic asthma	-	Significant association between atopic asthma and living in homes with either damage due to dampness (*p* < 0.05) or signs of mildew/mold (*p* = 0.06), and non-atopic asthma with natural gas home heating (*p* < 0.01).	Age, sex, persons per room, parental education
Renzi-Lomholt et al. [30]	Denmark	Cohort	29,851 children, median age: 8.0 years	Family socioeconomic position, metropolitan residence	Asthma	Asthma control, asthma severity, asthma exacerbations	Association between higher income and lower odds of poor asthma control (OR (odds ratio): 0.82; 95% CI: 0.72–0.93), severity (OR: 0.77; 95% CI: 0.63–0.94), and exacerbations (OR: 0.68; 95% CI: 0.58–0.79), higher family education with lower odds of asthma severity (OR: 0.82; 95% Cl: 0.72–0.93), exacerbations (OR: 0.84; 95% Cl: 0.72–0.98), and poor asthma control (OR: 0.82; 95% Cl: 0.73–0.93), metropolitan residence with higher odds of poor asthma control (OR: 1.07; 95% Cl: 1.00–1.15), asthma exacerbations (OR: 1.24; 95% Cl: 1.13–1.35), and asthma severity (OR: 1.13; 95% Cl: 1.01–1.27).	Age, sex
Rocco et al. [32]	Italy	Cross-sectional	2687 adolescents, 10–14 years	Parental education	Physician-diagnosed asthma, current asthma, current allergic rhinitis	-	Indirect effect of parental education on physician-diagnosed asthma, mediated by pregnancy maternal smoking (coefficient: 0.2350; *p* < 0.05), and current allergic rhinitis mediated by early environmental tobacco smoke (coefficient: 0.2002; *p* < 0.05).	Number of children in the family, people per room, parental smoking during pregnancy, environmental tobacco smoke exposure in early life, high residential traffic, mold/dampness in the child’s bedroom, pet ownership, obesity
Rodrigues et al. [33]	Portugal	Observational ecological	Not mentioned, 0–14 years	Air pollution (PM_10_)	Asthma	Hospital admissions	An increase in PM_10_ concentration led to a 2% increase in asthma hospital admissions.	Age, sex, season, long-term trend, calendar time
Rogerson et al. [58]	USA	Retrospective cohort	25,063 children, 2–18 years	SVI, household income, food access, transportation access	Asthma	Readmissions, hospitalizations	Significant association between high SVI and increased rate of asthma hospitalization (adjusted PAR: 1.09; 95% Cl: 1.03–1.15).	Age, sex, race/ethnicity, residence
Ryan et al. [59]	USA	Retrospective cohort	4849 children, <12 years	Redlining, community-level poverty, neighborhood socioeconomic position	Asthma	-	Association between SVI and high odds of asthma (OR: 1.10; 95% Cl: 1.01–1.19) and residing in Grade-D tracts with high odds of asthma (adjusted OR: 1.03; 95% Cl: 1.01–1.05), with 79% of this increase mediated by low-income households.	Sex, race/ethnicity, parental history of asthma, maternal education, maternal smoking during pregnancy, random intercept for census tract
Schreiber et al. [24]	Canada	Cross-sectional	86 children, mean age: 1.6 years	Indoor environmental quality (mold measurement), air quality	Eczema	Skin morbidity, annualized visits	An inverse association between annualized eczema visits and surface area of mold (RR: 0.14; 95% Cl: 0.01–0.93).	Age, sex, parental history of atopy, housing conditions, carbon dioxide levels, relative humidity, endotoxin concentration in dust
Shanahan et al. [60]	USA	Cross-sectional	831 children, mean age: 7.9 years	COI	Asthma	Current asthma, lung function	No associations between overall neighborhood COI scores and current asthma (adjusted OR: 0.93; 95% Cl: 0.77–1.14) or lung function (adjusted OR: 0.23 (−0.25 to 0.71).	Sex, race/ethnicity, maternal education, household income, maternal smoking during pregnancy, parental asthma history, census tract clustering
Sharma et al. [61]	USA	Case-crossover	14,5834 children and adolescents, 5–17 years	Neighborhood violence, SDI	Asthma	ED visits	Inverse association between SDI and asthma-related ED visits (*p* < 0.05). and asthma-related ED visits with lower levels of violence and deprivation communities (*p* < 0.05). Strong associations between PM_2.5_ and SO_2_ and asthma ED visits during the cold season on lag day 1, with increases of 4.90% (95% CI: 3.77–6.04) and 8.57% (95% CI: 5.99–11.21), respectively. In warm season, NO_2_ and O_3_: stronger effects on asthma ED visits on lag days 1 (7.86%; 95% CI: 6.66–9.07) and 2 (4.75%; 95% CI: 3.53–5.97), respectively.	Age, sex, race/ethnicity, environmental exposures (air pollution), relatively humidity, stratification by season
Siegfried et al. [35]	USA	Retrospective cohort	268,580 children with Medicaid insurance, mean age: 5.1 years, and 338,678 children with commercial insurance, mean age: 5.6 years	Healthcare access (insurance)	Atopic dermatitis	Healthcare utilization	A high EDR (defined as at least 33% of ambulatory visits occurring in the emergency department) was observed among Medicaid patients compared to those with commercial insurance (9.3% versus 3.2%, respectively, *p* < 0.001).	Age, sex, type of provider seen on the index visit, AD-related comorbidities
Telzak et al. [62]	USA	Cross-sectional	4887 children, mean age: 12.2 years	Unmet social needs	Persistent asthma	Asthma severity	Significant associations between food insecurity and persistent asthma severity status (*p* = 0.03), healthcare-related transportation, and persistent asthma severity status (*p* < 0.001) in the unadjusted analysis, persistent asthma severity status with housing quality (*p* = 0.04), and housing instability (*p* = 0.04).	Age, sex, race/ethnicity, insurance status, preferred language (English vs. non-English)
Titus et al. [63]	USA	Cohort	108,969 children and adolescents, 0–15 years	Housing type, housing age, neighborhood poverty	Asthma	Asthma prevalence	High asthma prevalence among children living in public or other subsidized housing (17.3% and 18.1% for non-Hispanic Black and Hispanic children, respectively).	Age, sex, race/ethnicity
Tyris et al. [64]	USA	Cross-sectional	4321 children, 2–17 years	Educational attainment, vacant housing, violent crime, living in poverty	Asthma	ED encounters, hospitalizations	Significant associations between violent crime and increased ARR for ED encounters due to asthma (estimate: 35.3; 95% CI: 10.2–60.4; *p* = 0.006) and low educational attainment with ARR for asthma ED encounters (estimate: 12.1; 95% Cl: 8.4–15.8); *p* < 0.001) and asthma hospitalizations (estimate: 1.2; 95% Cl: 0.2–2.2; *p* = 0.016).	Age, sex, percent of prescribed controller asthma medication
Tyris et al. [65]	USA	Cross-sectional	8,049,695 children, median age: 11 years	Economic stability, education access and quality, healthcare access and quality, social and community context, neighborhood and built environment	Asthma	Healthcare utilization	Association between experiencing discrimination (adjusted OR: 3.26; 95% Cl: 1.75–6.08), being a victim of violence (adjusted OR: 2.11; 95% CI: 1.11–4.0), and receiving free or reduced lunch (adjusted OR: 2.16; 95% CI: 1.57–2.98) with highest odds of asthma-related healthcare utilization.	Age, sex, comorbidities
Wesley et al. [66]	USA	Ecological	1,999,718 children and adolescents, <18 years	Neighborhood racial composition, neighborhood poverty	Asthma	Asthma incidence, ED visits	Association between a 1% increase in the proportion of the population with a poverty ratio under 2.0 with a 3.42% increase in acute asthma incidence (IRR: 1.91; 95% CI: 1.43–2.56), high PM_2.5_ levels with more frequent asthma-related ED visits, and non-White children with higher asthma incidence (adjusted IRR: 4.80; 89% CI: 4.12–5.61).	Population < 18 years in each census tract
Wey et al. [26]	Nigeria	Cross-sectional	490 children, 6 months–14 years	Ethnicity, parental educational level	Atopic dermatitis	-	No significant association between AD and lowest maternal and paternal education (*p* = 0.688 and *p* = 0.136, respectively) and Nupe ethnicity, in multivariate analysis	Sex, ethnicity, religion
Yang-Huang et al. [34]	UK, the Netherlands, Sweden, Australia, USA, Canada	Prospective cohorts	31,210 children, 0–6 years	Maternal education, household income	Asthma	Ever had asthma, asthma exacerbations, medication control	Association between low household income and a higher risk ratio for ever asthma (RR: 1.28; 95% CI: 1.15–1.43), wheezing/asthma attacks (RR: 1.22; 95% CI: 1.03–1.44), and increased risk of asthma with medication control (RR: 1.25; 95% CI: 1.01–1.55). Association between low maternal education with a high-risk ratio of ever asthma (RR: 1.24; 95% Cl: 1.13–1.37), high risk of wheezing/asthma attack (RR: 1.14; 95% Cl: 0.97–1.35), and increased risk of asthma with medication control (RR: 1.16; 95% Cl: 0.97–1.40).	Age, sex, maternal age at birth, maternal ethnic background

SES: socioeconomic status; ED: emergency department; r: Pearson correlation coefficient test; F: F-statistic; R^2^: coefficient of determination; OR: odds ratio; Cl: confidence interval; vs.: versus; BMI: Body Mass Index; ARPR: at risk of poverty rate; COI: Childhood Opportunity Index; SVI: Social Vulnerability Index; PM_2.5_: particulate matter with aerodynamic diameter ≤ 2.5 μm; O_3_: ozone; sβ: standardized coefficient; PM_2.5–10_: with diameter 2.5/10 μm or less; PICU: pediatric intensive care unit; ICS: inhaled corticosteroids; LABA: long-acting beta agonist; PCP: primary care provider; Q: Quantile; AA: African American; ADI: Area Deprivation Index; HR: hazard ratio; AD: atopic dermatitis; PM_10_: particulate matter with aerodynamic diameter ≤ 10 μm; PAR: proportion attributable ratio; SDI: Socioeconomic Deprivation Index; RR: risk ratio; EDR: emergency department reliance; SO_2_: sulfur dioxide; ARR: at-risk-rate; NO_2_: nitric oxide; IRR: incidence rate ratio; * Estimates that approach significance with *p* < 0.001 [48].

### 3.3. Main Studies’ Findings

The most frequently studied domain of SDOHs was Neighborhood and Built Environment (n = 26), followed by Economic Stability (n = 24), Social and Community Context (n = 21), Healthcare Access and Quality (n = 12), and Education Access and Stability (n = 10) (Table 2) The most examined SDOH factors included discrimination (n = 12), parental education (n = 11), neighborhood and residential conditions (n = 10), housing quality (n = 9), household income (n = 8), and air pollution (n = 6). The vast majority of studies (n = 31) found positive associations between the examined SDOH factors and asthma and/or allergic disease outcomes or healthcare utilization. The most frequently evaluated SDOH with positive associations were neighborhood and residential conditions (n = 10), discrimination (n = 8), parental education (n = 7), housing quality (n = 6), air pollution (n = 6), and household income (n = 5). Concerning the disease evaluated, thirty-eight studies examined asthma, and most of them (n = 27) found a positive association between at least one SDOH and asthma outcomes and healthcare utilization.

The majority of the included studies were conducted in high-risk populations—pediatric groups who accumulate social or environmental disadvantages such as poverty, low caregiver education, minoritized racial/ethnic status, residence in highly deprived or polluted neighborhoods, sub-standard or unstable housing, and limited access—(n = 23); eleven studies were classified as population-based; the remaining studies were both population-based and conducted in high-risk populations (n = 10). Seven studies investigated AD, with the majority (n = 5) reporting a positive correlation [24,27,35,37,38]. Two studies focused on allergic rhinitis, revealing a positive association [32,38]. Finally, two studies addressed food allergy, and both reported positive associations [38,39].

Nine studies used composite SDOH measures [36,42,49,55,56,58,59,60,61]. Four studies assessed the Social Vulnerability Index (SVI) [42,49,58,59]. Children living in high SVI neighborhoods had a 9% higher proportion of asthma-related hospitalizations compared to those in low SVI areas (adjusted PAR (proportion attributable ratio): 1.09; 95% Cl (95% confidence interval): 1.03–1.15) [58]. In contrast, children with critical asthma, living in hot spot neighborhoods—defined as census tracts with a pediatric intensive care unit (PICU) asthma admission rate at or above the 90th percentile—had higher SVI [49]. High odds of asthma were linked to SVI (OR (odds ratio): 1.10; 95% Cl: 1.01–1.19) in the Ryan et al. study [59]. Conversely, another study found no association between SVI and asthma incidence [42]. One study evaluated the Socioeconomic Deprivation Index and noted an inverse association with asthma-related ED visits (*p* < 0.05) [61]. Three studies used the Childhood Opportunity Index (COI) [42,49,60]. Children with critical asthma living in hot spot neighborhoods had lower COI (*p* < 0.05) [49]; meanwhile, high and very high COI in early life, compared to very low COI, was related to lower asthma incidence (adjusted IRR (incidence rate ratio): 0.87; 95%CI: 0.75–1.00) [42]. Conversely, Shanahan et al. found no associations between overall neighborhood COI scores and current asthma (adjusted OR: 0.93; 95% Cl: 0.77–1.14) or lung function (adjusted OR: 0.23; 95% Cl: −0.25 to 0.71) [60]. One study examined the Area Deprivation Index (ADI) and noted no significant associations with asthma readmissions (*p* > 0.05) [56], whilst a cross-sectional study detected no association between the Deprivation Index (DI) and AD severity [36]. Additionally, in Mersha et al., socioeconomic hardship—measured as a composite of low income, limited caregiver education, public insurance, non-homeownership, lack of vehicle access, and single-parent status—was significantly associated with asthma readmission (*p* = 0.02) and partially mediated the effect of African ancestry on readmission risk (*p* < 0.001) [55].

#### 3.3.1. Economic Stability

##### Household Income

Eight studies examined the impact of household income on asthma and/or allergic disease outcomes and healthcare utilization [25,27,30,31,34,36,38,59]. Most studies found a positive association between household income and disease outcomes or healthcare utilization [27,30,34,38,59]. Yang-Huang et al. reported that low household income was associated with a higher risk ratio (RR) for ever asthma (RR: 1.28; 95% CI: 1.15–1.43) in children aged 0–6 years, wheezing/asthma attacks (RR: 1.22; 95% CI: 1.03–1.44), and increased risk of asthma with medication control (RR: 1.25; 95% CI: 1.01–1.55) [34]. Positive associations between low and median household income levels and asthma were observed in Ryan et al., with a 16% increase in asthma odds per standard deviation increase in low-income households (OR: 1.16; 95% CI: 1.08–1.24) [59]. Additionally, the study of Renzi-Lomholt et al. showed that higher income was linked to lower odds of poor asthma control (OR: 0.82; 95% CI: 0.72–0.93), severity (OR: 0.77; 95% CI: 0.63–0.94), and exacerbations (OR: 0.68; 95% CI: 0.58–0.79), suggesting that low income was related to higher odds of poor asthma control, exacerbations, and severe asthma [30]. Kim et al. found that children in the highest income quartile (Quantile 4, Q4: highest) had greater healthcare utilization for allergic asthma (OR: 1.36; 95% CI: 1.08–1.71; *p* < 0.001) and AD (OR: 1.58; 95% CI: 1.41–1.76; *p* < 0.001) compared to other income groups (Q1–Q3, Q1: lowest, Q2: middle, Q3: high). These findings were attributed to the financial barriers faced by lower-income groups, which can limit access to medical care [27]. Le et al. found a significant association between financial instability and food allergy prevalence in Asian Indian children (*p* = 0.02) [38].

Conversely, Caffrey Osvald et al. documented no association between the lowest maternal income and current asthma at 5 years compared to the highest maternal income (adjusted OR: 0.98; 95% CI: 0.94–1.02) [31], while Joy et al. reported that socioeconomic status, including income, was not associated with asthma control (*p* = 0.95) [25]. Similarly, Reimer-Taschenbrecker et al. found no correlation between income and the odds of moderate-to-severe AD, compared to mild AD. For example, children from households earning USD 25,000–USD 49,999 had an OR of 0.80 (95% CI: 0.27–2.36) compared to those with income over USD 100,000, with all confidence intervals crossing 1.0 [36].

##### Poverty

Two studies examined the association between poverty and asthma outcomes and healthcare utilization, with mixed findings [29,66]. Wesley et al. reported that a 1% increase in the proportion of the population with a poverty ratio under 2.0 was associated with a 3.42% increase in acute asthma incidence (IRR: 1.91; 95% CI: 1.43–2.56) [66]. In contrast, a cross-sectional study, involving children and adolescents aged 6–14 years, found no association between the poverty risk rate tertile and asthma control (*p* = 0.092) [29].

##### Food Insecurity

Two studies that evaluated the correlation between food insecurity and asthma outcomes and healthcare utilization revealed a positive association [62,65]. Telzak et al. reported a significant relationship between food insecurity and persistent asthma severity (*p* = 0.03) [62]. Tyris et al. found that receiving free or reduced cost lunches was associated with increased healthcare utilization due to asthma (adjusted OR: 2.16; 95% CI: 1.57–2.98) [65].

#### 3.3.2. Education Access and Quality

##### Parental Educational Attainment

Eleven studies evaluated the impact of parental educational attainment on asthma or allergic disease outcomes and healthcare utilization. Most of these studies reported a positive association (n = 7) [28,29,30,32,34,38,64]. A cross-sectional study demonstrated that medium/high levels of parental educational attainment were associated with a reduced risk of unscheduled or urgent asthma visits (maternal education attainment—OR: 0.50; 95% Cl: 0.27–0.95; *p* = 0.034 and paternal education attainment—OR: 0.51; 95% Cl: 0.28–0.94; *p* = 0.030) [29]. Similarly, higher family education status was linked to lower odds of asthma severity (OR: 0.82; 95% Cl: 0.72–0.93), exacerbations (OR: 0.84; 95% Cl: 0.72–0.98), and poor asthma control (OR: 0.82; 95% Cl: 0.73–0.93) [30]. Conversely, low educational attainment was correlated with increased rates of asthma-related ED encounters (estimate: 12.1; 95% Cl: 8.4–15.8; *p* < 0.001) and asthma hospitalizations (estimate: 1.2; 95% Cl: 0.2–2.2; *p* = 0.016) [64]. Yang-Huang et al. reported that low maternal education was associated with elevated risk ratios for ever having asthma (RR: 1.24; 95% Cl: 1.13–1.37), wheezing/asthma attack (RR: 1.14; 95% Cl: 0.97–1.35), and asthma requiring medication control (RR: 1.16; 95% Cl: 0.97–1.40) [34]. Another cross-sectional study found that adolescents whose parents possessed only primary or secondary education and were exposed to indoor dampness or mold exhibited nearly twice the likelihood of having a history of asthma or current asthma symptoms compared to unexposed peers. Moreover, higher parental education was associated with approximately 50% lower odds of indoor dampness or mold exposure and asthma compared to primary or secondary education (adjusted OR: 1.55; 95% Cl: 1.04–2.32 and adjusted OR: 1.96; 95% Cl: 1.06–2.19, respectively) [28]. Le et al. noted a significant association between lower education level with hay fever and respiratory allergy for non-Hispanic “other Asian” American children (*p* = 0.001 and *p* = 0.006, respectively) [38]. Additionally, an indirect effect of parental education on physician-diagnosed asthma was observed, mediated by maternal smoking (coefficient: 0.2350; *p* < 0.05), and on current allergic rhinitis mediated by early environmental tobacco smoke (coefficient: 0.2002; *p* < 0.05) [32].

Conversely, four studies documented weak or nonsignificant associations between parental educational attainment and asthma or allergic disease outcomes [25,26,31,36]. For example, Caffrey Osvald et al. found a weak association between the lowest maternal education level and current asthma at age 5 compared to the highest level (adjusted OR: 0.98; 95% Cl: 0.94–1.02) [31]. Joy et al. reported no significant association between maternal education and asthma control (*p* = 0.76) [25]. In the multivariate analysis in the Wey et al. study, AD was not significantly related to the lowest maternal and paternal education (*p* = 0.688 and *p* = 0.136, respectively) [26]. Moreover, parental education was not correlated with the odds of moderate-to-severe versus mild AD in another study. For example, compared to children whose parents had a graduate degree, those whose parents had only some college education had an OR of approximately 0.82 (95% CI: 0.28–2.36), with all confidence intervals crossing 1.0 [36].

#### 3.3.3. Healthcare Access and Quality

Four studies examined the relationship between healthcare access, asthma or allergic disease outcomes, and healthcare utilization. Each study reported a significant positive association [35,38,46,62]. Telzak et al. identified a statistically significant association between healthcare-related transportation and persistent asthma severity status (*p* < 0.001), in the unadjusted analysis [62]. Siegfried et al. compared healthcare utilization patterns in commercially insured and Medicaid-insured children with AD, noting that the mean ED Reliance (EDR) was significantly higher for Medicaid-insured children (12%), compared to those with commercial insurance (6.3%) [35]. Moreover, a high EDR—defined as at least 33% of ambulatory visits occurring in the ED—was more prevalent among Medicaid patients than those with commercial insurance (9.3% versus 3.2%, respectively, *p* < 0.001) [35]. Le et al. reported that limited access to healthcare was associated with a higher likelihood of skin allergies in Chinese children (17.3% vs. 10.2%; 95% CI: 5.4–29.1% vs. 2.3–18.1%), hay fever in Asian Indian children (7.3% vs. 4.5%; 95% CI: 1.9–12.6% vs. 0.6–8.5%), and a higher predicted probability of any allergic disease in both subgroups, though the confidence intervals overlapped in some cases [38]. At the same time, Correa-Agudelo et al. demonstrated that children under 18 years with Medicaid insurance experienced a 7% higher rate of ED visits due to asthma compared to those with commercial insurance (1.07; 95% Cl: 1.03–1.1) [46].

#### 3.3.4. Neighborhood and Built Environment

##### Neighborhood and Residential Conditions

Ten studies evaluated the impact of environmental and residential conditions on asthma and/or allergic disease outcomes and healthcare utilization, reporting consistent positive associations [30,40,43,45,49,50,53,56,57,59]. Renzi-Lomholt et al. noted that metropolitan residence was associated with increased odds of poor asthma control (OR: 1.07; 95% Cl: 1.00–1.15), asthma exacerbations (OR: 1.24; 95% Cl: 1.13–1.35), and asthma severity (OR: 1.13; 95% Cl: 1.01–1.27) [30]. Children residing in Grade-D tracts—designated as “high-risk” or “hazardous” areas—exhibited higher odds of asthma (adjusted OR: 1.03; 95% Cl: 1.01–1.05), with 79% of this increase mediated by low-income household status [59]. Hauptman et al. demonstrated that proximity to major roadways was significantly associated with an increased number of asthma symptom days (*p* < 0.01). Conversely, beyond a distance of 100 m from a major roadway, children had a 29% lower likelihood of experiencing a symptom day in the past two weeks for every additional 100 m increase in distance (OR 0.71; 95% CI: 0.58–0.87; *p* < 0.01) [50]. Additionally, children residing farther from major roadways reported significantly lower healthcare utilization (OR 0.63; 95% CI 0.47–0.85; *p* < 0.01) and were less likely to experience poor asthma control (OR 0.80; 95% CI 0.69–0.94; *p* < 0.01) [50]. Aryee et al. documented that children living in neighborhoods with high levels of social support, safety, and quality had reduced asthma prevalence (OR: 0.9; 95% Cl: 0.8–1.0; *p* = 0.02, OR: 0.7; 95% Cl: 0.5–0.9; *p* = 0.02 and OR: 0.9; 95% Cl: 0.8–1.0; *p* = 0.03, respectively) [43]. Children residing in high-density rental areas were 2.33 times more likely to experience asthma exacerbations compared to those in newer, lower-density areas (adjusted OR: 2.33; 95% CI: 1.25–4.44) [53]. Adams and Knuth et al. showed that neighborhoods with the lowest median income, such as the Bronx and Harlem, reported the highest asthma ED rates, with Harlem’s ZIP code 10037 reaching 597.2 ED visits per 10,000 children [40]. Pollack et al. found that children living in high-poverty neighborhoods had at least one exacerbation per three-month period. However, after relocating to low-poverty areas, the exacerbation rate decreased to 8.5% (adjusted difference: −6.8%; 95% CI: −11.9% to −1.7%; *p* = 0.009) [57]. Furthermore, the number of symptom days declined from 5.1 days to 2.7 days in the past week (adjusted difference: −2.37 days; 95% CI: −3.14 to −1.59; *p* < 0.001) [57]. Molina et al. reported a significant association between increasing residential instability and adverse asthma outcomes (more severe asthma (OR: 1.18; 95% Cl: 1.05–1.32; *p* = 0.004); higher risk of 365-day ED readmission (HR (hazard ratio): 1.10; 95% Cl: 1.05–1.15; *p* < 0.001); and higher risk of 365-day re-hospitalization (HR: 1.09; 95% Cl: 1.03–1.14; *p* = 0.002) [56]. Commodore et al. noticed that children exposed to high neighborhood traffic density had higher odds of experiencing asthma-like symptoms compared to those unexposed (adjusted OR: 2.1; 95% Cl: 1.12–3.62) [45]. Additionally, children living in hot spot neighborhoods had significantly greater social vulnerability (SVI: 0.67; interquartile range (IQR): 0.49–0.87) and lower childhood opportunity (COI: 17; IQR: 7–43) than those outside hot spots (SVI: 0.46; IQR: 0.24–0.73 and COI: 48; IQR: 24–75; *p* < 0.0001). These differences were associated with greater healthcare use, including more inpatient bed days (14.8 vs. 8.8; *p* < 0.0001) and higher bed day rates per 1000 children (13.0 vs. 5.0; *p* < 0.0001), despite no difference in readmission rates [49].

##### Housing Quality

Nine studies examined the relationship between housing quality and asthma or allergic disease outcomes, as well as healthcare utilization. Most studies (n = 6) reported a positive association [23,28,38,48,62,63]. Antonogeorgos et al. showed that current exposure to indoor dampness and/or mold was significantly associated with both ever had asthma symptoms (adjusted OR: 1.52; 95% Cl: 1.06–2.19; *p* < 0.001) and current asthma (adjusted OR: 1.66; 95% Cl: 1.1–2.51; *p* < 0.001) [28]. Poor housing quality, characterized by elevated levels of airborne and bedroom floor mouse allergen concentrations, was associated with air trapping but not with airflow limitation (OR: 1.19; 95% Cl:1.02–1.37; *p* = 0.02 for each 2-fold increase in airborne mouse allergen; OR: 1.23; 95% Cl: 1.07–1.41; *p* = 0.003 for each 2-fold increase in bedroom floor mouse allergen). Other indoor exposures—such as cockroach (*p* = 0.55), dog (*p* = 0.37), cat (*p* = 0.66), particulate matter (*p* = 0.73 for PM_2_._5_: particulate matter with aerodynamic diameter ≤ 2.5 μm; *p* = 0.55 for PM_2_._5–10_: with diameter 2.5/10 μm or less), and nicotine (*p* = 0.81)—were not significantly associated with air trapping or airflow limitation [48]. Rennie et al. found that atopic asthma was correlated with signs of household dampness damage (*p* < 0.05) and presence of mildew or mold (*p* = 0.06), whereas non-atopic asthma was significantly associated with natural gas heating systems (*p* < 0.01) [23]. Telzak et al. identified significant associations between persistent asthma severity and both poor housing quality (*p* = 0.04) and housing instability (*p* = 0.04) [62]. Elevated asthma prevalence was reported among children residing in public or subsidized housing (17.3% and 18.1% for non-Hispanic Black and Hispanic children, respectively) [63]. Furthermore, Le et al. observed a significant association between alternative housing arrangements (e.g., rent) and skin allergy in non-Hispanic Filipino children (*p* = 0.01) [38].

Conversely, Schreiber et al. identified an inverse association between annual eczema-related healthcare visits and the surface area of mold exposure (RR: 0.14; 95% Cl: 0.01–0.93) [24]. Jung et al. reported mixed findings: while home and school PM_2_._5_ exposure was not associated with asthma severity overall, among children living in redlined neighborhoods, higher PM_2_._5_ exposure correlated with increased asthma severity (*p* < 0.005) [52]. No significant association was found between home and school pollutants and lung function or asthma severity in children living in non-redlined neighborhoods (*p* > 0.005) [52].

One study showed no significant association. Housing insecurity—measured by recent address change—was not significantly related to asthma-specific 30- or 90-day revisit rates (*p* = 0.114) [47].

##### Violence and Crime

Three studies examined the impact of violence and crime on asthma outcomes and healthcare utilization [61,64,65]. No studies assessing other allergic diseases examined this association. Two studies noted significant positive associations [64,65]. Violent crime was associated with increased at-risk rates (ARRs) for ED encounters related to asthma (estimate: 35.3; 95%CI: 10.2–60.4; *p* = 0.006) [64]. Additionally, the highest adjusted OR for asthma healthcare utilization was observed among individuals identified as victims of violence (adjusted OR: 2.11; 95%CI: 1.11–4.0) [65]. However, Sharma et al. reported an inverse association, noting that communities characterized by lower levels of violence and deprivation exhibited significantly fewer asthma-related ED visits (*p* < 0.05) [61].

##### Air Pollution

Six studies evaluated the impact of air pollution on asthma healthcare utilization and identified positive associations [33,44,46,51,61,66]. No studies examining other allergic diseases have explored this relationship. Baek et al. demonstrated that elevated average ozone (O_3_) levels in children’s residential neighborhoods were significantly linked to increased asthma-related hospitalizations (OR: 1.78; 95% CI: 1.01–3.14; *p* = 0.045) [44]. In the study of Correa-Agudelo et al., levels of PM_2_._5_, pollen, and outdoor mold exposure were related to higher rates of asthma-related ED visits among both European American and African American children (*p* < 0.001) [46]. Increased air pollution was also associated with more frequent asthma exacerbations—specifically exposure to PM_2_._5_ during both warm and cold months (OR: 1.05; 95% Cl: 1.02–1.07 and OR: 1.03; 95% Cl: 0.98–1.08, respectively) and O_3_ during cold months (OR: 1.08; 95% Cl: 1.02–1.14) [51]. In the study by Rodrigues et al., rising PM_10_ concentrations led to a 2% increase in asthma hospital admissions [29]. Additionally, high PM_2_._5_ levels were correlated with more frequent asthma-related ED visits; for example, census tracts in the highest PM_2_._5_ quintile had over twice the incidence rate compared to the lowest (IRR: 2.38; 89% CI: 1.60–3.56) [66]. Sharma et al. found strong associations between PM_2_._5_ and sulfur dioxide (SO_2_) concentrations and asthma ED visits during the cold season on lag day 1, with respective increases of 4.90% (95% CI: 3.77–6.04) and 8.57% (95% CI: 5.99–11.21). In the warm season, nitric dioxide (NO_2_) and O_3_ exerted stronger effects on asthma ED visits on lag days 1 (7.86%; 95% CI: 6.66–9.07) and 2 (4.75%; 95% CI: 3.53–5.97) [61].

#### 3.3.5. Social and Community Context

##### Discrimination

Twelve studies investigated the effect of discrimination on asthma and/or allergic disease outcomes and healthcare utilization. Most of the studies presented significantly positive associations (n = 8) [37,38,39,40,41,54,65,66]. Tyris et al. reported that experiences of discrimination were associated with significantly higher odds of asthma-related healthcare utilization (adjusted OR: 3.26; 95% Cl: 1.75–6.08) [65]. Adams and Knuth identified a significant positive correlation between asthma-related ED visits and the percentage of Black children (r(35): 0.41; *p* < 0.05), whereas a negative correlation was found for White residents (r(35): −0.44; *p* < 0.01). The percentage of non-Hispanic Black residents and ambient air temperatures were statistically significant predictors of asthma-related ED visit rates (F(3,38): 22.354; *p* < 0.001) [40]. Aratani et al. showed that among English-speaking families, Black individuals were less likely to be hospitalized during their first asthma ED visit (OR: 0.787; 95% CI: 0.715–0.866) but more likely to return to the ED (OR: 1.291; 95% CI: 1.205–1.383) compared to White individuals. In contrast, English-speaking Asian/Pacific Islanders had a higher likelihood of hospitalization (OR: 2.150; 95% CI: 1.827–2.530) compared to White individuals. Among non-English speaking families, Hispanic and Asian/Pacific islanders were also more likely to be hospitalized during their first asthma ED visit (OR: 1.427; 95% Cl: 1.332–1.529 and OR: 1.605; 95% Cl: 1.213–2.124, respectively), while all non–English-speaking groups were less likely to revisit the ED compared to English-speaking Whites [41]. In the study by Kim et al., African American children had higher ratios of asthma ED visits to outpatient visits (OR: 1.32; 95% CI: 1.08–1.62) and asthma hospitalizations to outpatient visits (OR: 1.50; 95% CI: 1.30–1.73). They also exhibited higher ratios of ED visits (OR: 1.36, 95% CI: 1.10–1.68) and hospitalizations (OR: 1.47; 95% CI: 1.26–1.71) compared to visits to primary care physicians [54]. In the study by Wesley et al., non-White children had significantly higher asthma incidence (adjusted IRR: 4.80; 89% CI: 4.12–5.61) [66]. Mahdavinia et al. reported increased odds of finfish allergy (adjusted OR: 2.54; *p* < 0.01), and shellfish allergy (adjusted OR: 3.10; *p* < 0.001), among African American children, along with a significantly higher asthma prevalence compared to White children (asthma prevalence of 60.5% in African Americans and 27.2% in Whites, OR: 2.70, *p* < 0.001) [39]. Furthermore, Choragudi et al. found that White children with eczema experienced a greater annual increase in well-child checkups and a rising trend in medical specialist visits, unlike other minority groups, whose trends remained static [37]. Le et al. showed that the number of unfavorable SDOHs was lowest among non-Hispanic Asian Indian and Chinese children (mean: 0.7) and highest among non-Hispanic “other Asian” American children (mean: 1.2) [38].

In contrast, four studies documented no significant associations [26,36,46]. Correa-Agudelo et al. found no correlation between discrimination and asthma ED visits (sβ (standardized coefficient): 0.006; *p* = 0.796) [46]. In the study by Wey et al., the Nupe ethnic group showed no significant association with atopic dermatitis (*p* = 0.051) in multivariate analysis [26]. Similarly, Reimer–Taschenbrecker et al. found no correlation between discrimination and the odds of moderate-to-severe AD, compared to mild AD [36]. For example, the odds of moderate-to-severe disease in Black children were OR: 1.24; 95% CI: 0.53–2.92, and in Asian children OR: 1.36; 95% CI: 0.50–3.70, with all confidence intervals crossing 1.0 [36]. Ryan et al. did not observe a substantial overall effect of historical redlining on asthma prevalence (adjusted OR: 1.03; 95% CI: 1.01–1.05), with 79% of the effect mediated by neighborhood poverty, suggesting no substantial direct effect of redlining alone on asthma prevalence. However, children residing in Grade-D neighborhoods—areas historically subjected to redlining—demonstrated elevated odds of asthma (adjusted OR: 1.03; 95% CI: 1.01–1.05), with 79% of this effect mediated by low household income [59].

### 3.4. Risk of Bias

Based on the quality assessment of the included studies using validated tools (Newcastle–Ottawa Scale [19], AXIS [20], and adapted tools for ecological [21] and case-crossover designs [22]), the overall methodological quality was rated as good for the majority of studies (Tables S1–S4 in the Supplement). Specifically, most cohort studies (n = 19) scored between 8 and 9 out of 9 stars, indicating low risk of bias. The remaining two cohort studies were assessed as being of fair quality, primarily due to limitations in exposure assessment and control for confounders [45], or incomplete adjustment for confounding and limited follow-up detail [39]. Similarly, all cross-sectional studies assessed using the AXIS tool achieved scores between 15 and 17 out of 20, also suggesting good quality. The ecological studies by Adams and Knuth [40], Rodrigues et al. [33], and Wesley et al. [66], and the case-crossover studies by Huang et al. [51] and Sharma et al. [61] were likewise judged to be of good quality with low risk of bias. Table 3 represents the overall risk of bias of the selected studies.

## 4. Discussion

In this systematic review, we synthesized evidence on the influence of multilevel SDOHs on asthma and allergic disease outcomes and healthcare utilization in children and adolescents, with a specific focus on how these determinants generate or widen health inequities. The most frequently studied domain of SDOHs was Neighborhood and Built Environment (n = 26), followed by Economic Stability (n = 24), Social and Community Context (n = 21), Healthcare Access and Quality (n = 12), and Education Access and Stability (n = 10). Most studies reported positive associations between the examined SDOHs and both disease outcomes and healthcare utilization. The most frequently evaluated SDOHs with positive associations were neighborhood and residential conditions, discrimination, parental education, housing quality, air pollution, and household income. Regarding the disease evaluated, the majority of studies on asthma demonstrated a positive association with at least one SDOH, as did those investigating AD. Furthermore, a limited number of studies on allergic rhinitis and food allergy also identified positive associations.

Several of our findings align with previous systematic reviews and meta-analyses. For instance, Tyris et al. conducted a U.S.-based systematic review examining SDOHs and pediatric asthma healthcare utilization across geographic regions and identified key influences including healthcare and primary care access, poverty, education, discrimination, crime, housing quality, and environmental conditions—many of which correspond with the determinants highlighted in our review [11]. Another review found that Neighborhood and Built Environment and Social and Community Context were the most influential SDOH domains affecting pediatric asthma exacerbations [12], consistent with our finding that Neighborhood and Built Environment was the most frequently studied domain, with neighborhood and residential conditions being the most commonly associated factors. Furthermore, a meta-analysis, assessing the impact of SDOHs on ED outcomes in pediatric populations, found that SES and discrimination were the most frequently reported SDOHs, along with low income, neighborhood deprivation, public insurance, and proximity to ED. It also noted that children from racial and ethnic minority groups, particularly Black and Latino children, had higher ED utilization rates, longer wait times, and lower odds of hospital admission following ED visits [67]. Although that meta-analysis examined a broader range of pediatric conditions, its findings align with our results, which indicated that Black children, non-White populations, and other minority groups had higher odds of asthma/allergic diseases incidence, ED visits, and hospitalizations [38,40,41,54,66].

Compared to our extensive understanding of SDOHs in asthma, there is considerably less research available on allergic and immunologic diseases as covered in previous reviews and rostrums [68]. Similarly, in our review, most of the included studies focused on asthma. Nevertheless, the undeniable impact of SDOHs on childhood allergic diseases persists, highlighting the urgent need for more studies that investigate the influence of SDOHs on outcomes and healthcare utilization related to allergic diseases. Le et al. correlated financial instability, poor housing conditions, lower parental education, and inaccessible care with an increased likelihood of allergic diseases in children, while also highlighting racial and ethnic disparities [38].

A few studies in our systematic review reported no significant associations between SDOHs and asthma and/or allergic disease outcomes or healthcare utilization, while others presented mixed findings. The lack of association observed in some studies may be attributable to limited statistical power, stemming from small sample sizes [60]. Conversely, another study suggests that this absence of association may be better explained by variations in study design and differences in how SDOHs were measured, rather than sample size limitations [25]. Additionally, the impact of certain SDOH factors may vary geographically, much like the regional variability seen in disease prevalence, further emphasizing the need for research across diverse populations and settings [36]. Another plausible explanation is that the observed associations are mediated by adversity and its interplay with other exposure factors. For instance, minority pediatric patients may exhibit higher healthcare utilization not due to genetic predisposition, but rather as a consequence of social and environmental exposures [55]. Furthermore, several studies (n = 9) employed composite SDOH measures, integrating multiple SDOH factors or domains. However, in some cases, results were not disaggregated to assess the independent contribution of each SDOH factor. This limited the ability to identify specific drivers of outcome variability, a concern also raised in a prior systematic review [11].

To enhance the interpretation of our findings, incorporating theoretical frameworks can be particularly valuable. The WHO Conceptual Framework for Action on the SDOHs provides a comprehensive approach to understanding the mechanisms through which SDOHs influence health outcomes [1]. According to this model, structural determinants—such as income, education, and social policies—act through intermediary determinants like material living conditions (e.g., poor housing quality), psychosocial stress, and limited access to healthcare. For example, poor housing quality—marked by dampness, mold, and pest infestations—can have a serious impact on respiratory health, especially in children. Such indoor conditions can cause airway inflammation, heightening the likelihood of asthma symptoms, exacerbations, and poorer disease control [69,70]. Mold exposure can provoke asthma symptoms through specific mechanisms. These include the unique properties of specific fungal species, the action of mold-derived proteases that compromise the integrity of the respiratory epithelial barrier, and the type 2-based inflammatory response triggered by the host’s immune system [71]. Additionally, children from low-income families face barriers to healthcare resources, leading to underutilization of asthma preventative treatments and poor medication adherence [72,73]. This, in turn, increases the risk of asthma exacerbations, hospitalizations, and the need for treatment of severe symptoms [7]. Moreover, experiencing racial discrimination can result in chronic psychological stress [74], which activates the hypothalamic–pituitary–adrenal (HPA) axis and the sympathetic nervous system, which in turn disrupts immune regulation, increasing inflammation and allergic responses [75].

Beyond the adverse SDOHs that dominate the literature, our review also uncovered protective or null associations—notably higher parental education, greater neighborhood greenness, and a high COI—highlighting that SDOHs place children on a continuum of vulnerability-to-resilience rather than acting as strictly harmful factors. Still, most included studies originate from urban U.S. settings, which limits the generalizability of our conclusions to other socioeconomic and healthcare system contexts. Because structural exposures such as poverty, systemic discrimination, and residential segregation cannot easily be randomized, the strongest available evidence for many SDOHs will continue to come from natural experiments and systematic reviews or meta-analyses of observational studies.

Observed racial and ethnic disparities in outcomes are interpreted not as innate biological differences, but as manifestations of the unequal distribution of adverse SDOHs across population groups. Structural racism funnels poverty, substandard housing, and pollution into minoritized neighborhoods, intensifying immune- and airway-damaging exposures [76]. Conceptual frameworks such as the “fundamental-cause” theory explain why upstream social conditions continue to reproduce health gaps across many diseases and over time [77]. Hence, race chiefly proxies cumulative adverse SDOHs; narrowing disparities will require structural-level policy reforms rather than race-specific biological searches [78].

The complex and multifactorial impact of SDOHs on diseases such as asthma and other allergic conditions necessitates the development of coordinated and multi-level interventions to mitigate their effects on disease outcomes or healthcare utilization. A meta-analysis addressing the relationship between social risk-based interventions for pediatric asthma and asthma-related healthcare utilization outcomes noticed that integrated approaches—including place-based asthma education, home environment remediation, and peer support-reduced asthma-related ED visits and hospitalizations among children [79]. Public health interventions that target improvements in nutrition, socioeconomic equity, education, housing environment, and healthcare infrastructure are essential for the prevention and management of childhood respiratory diseases such as asthma [80], as well as other allergic diseases. Moreover, it is crucial to communicate to both the public and policymakers that the consequences of childhood deprivation have lasting effects across the lifespan and can be transmitted to future generations [80].

Our findings underscore the significant overlap between pediatric asthma and allergic diseases, which complicates efforts to isolate these conditions in the literature. This overlap is particularly pronounced in younger age groups, where comorbid diagnoses are frequent and often analyzed together. Furthermore, the interpretation of findings should take into account regional and ethnic heterogeneity. For instance, many U.S.-based studies report disparities largely structured along White and non-White children, shaped by specific structural and socioeconomic factors. These patterns may not hold in non-U.S. contexts, where ethnic composition, social stratification, and healthcare access differ markedly. As such, extrapolation of findings across regions should be made with caution.

The present systematic review has several limitations. A key limitation of this review is the absence of prospective protocol registration (e.g., PROSPERO). When a review protocol is not publicly registered, there is a higher risk of undisclosed post-hoc methodological changes and selective outcome reporting, which may, in turn, exacerbate publication bias [18]. Another limitation is the heterogeneity across included studies, particularly in terms of sample sizes—ranging from 66 participants [25] to 8,049,695 children and adolescents [65]—as well as variations in age groups, the range of SDOHs examined, methodology assessment, confounding and covariate variables, and study designs. In addition, the vast majority of included studies were observational (cross-sectional and cohort studies) and lacked interventional components, making them more vulnerable to selection and confounding bias. As for the study design, ecological studies, included in this review, contributed valuable area-level insights but are more vulnerable to confounding and the ecological fallacy; their findings were therefore interpreted as hypothesis-generating and weighed accordingly in our synthesis. Finally, the generalizability of our findings may be limited due to the predominance of U.S.-based studies (n = 32), which reflect specific socioeconomic, environmental, and cultural contexts that may not be applied to other populations or healthcare systems [81].

## 5. Conclusions

In conclusion, this systematic review demonstrates that SDOHs significantly influence pediatric asthma and allergic disease outcomes, as well as healthcare utilization. The majority of studies included revealed positive associations between factors such as poor housing quality, low household income, limited parental education, and adverse neighborhood conditions with increased disease prevalence, higher exacerbation rates, and greater use of emergency healthcare services. These findings underscore the urgent need for coordinated, multi-level interventions and policies aimed at addressing underlying social, environmental, and economic factors. Such efforts are essential for reducing health disparities and improving clinical outcomes for vulnerable pediatric populations. Furthermore, they emphasize the importance of conducting well-designed studies to generate robust and causal evidence.

## Figures and Tables

**Table 2:** Classification of studies based on Healthy People 2030 Initiative—Social Determinants of Health.

Study	Economic Stability	Education Access and Quality	Health Care Access and Quality	Neighborhood and Built Environment	Social and Community Context
Adams and Knuth [40]				✓	✓
Antonogeorgos et al. [28]					✓
Antoñón et al. [29]	✓				✓
Aratani et al. [41]	✓				
Aris et al. [42]			✓	✓	
Aryee et al. [43]					✓
Baek et al. [44]			✓		✓
Caffrey Osvald et al. [31]			✓	✓	
Choragudi et al. [37]					✓
Commodore et al. [45]	✓			✓	✓
Correa-Agudelo et al. [46]	✓			✓	✓
Faison et al. [47]					
Grant et al. [48]	✓			✓	
Grunwell et al. [49]			✓	✓	✓
Hauptman et al. [50]	✓			✓	
Huang et al. [51]	✓			✓	✓
Joy et al. [25]	✓				
Jung et al. [52]				✓	✓
Khan et al. [53]	✓			✓	✓
Kim et al. [54]	✓		✓	✓	
Kim et al. [27]	✓	✓	✓		
Le et al. [38]	✓	✓	✓	✓	
Mahdavinia et al. [39]	✓				✓
Mersha et al. [55]	✓			✓	
Molina et al. [56]				✓	
Pollack et al. [57]	✓			✓	✓
Reimer-Taschenbrecker et al. [36]	✓		✓		✓
Rennie et al. [23]		✓		✓	
Renzi-Lomholt et al. [30]	✓	✓			
Rocco et al. [32]		✓			
Rodrigues et al. [33]				✓	
Rogerson et al. [58]	✓			✓	✓
Ryan et al. [59]	✓	✓	✓	✓	✓
Schreiber et al. [24]					✓
Siegfried et al. [35]			✓		
Shanahan et al. [60]				✓	
Sharma et al. [61]				✓	✓
Telzak et al. [62]	✓		✓	✓	
Titus et al. [63]	✓			✓	
Tyris et al. [64]		✓		✓	
Tyris et al. [65]	✓	✓	✓	✓	✓
Wesley et al. [66]	✓				✓
Wey et al. [26]		✓			
Yang-Huang et al. [34]	✓	✓			

**Table 3:** Overall risk of bias of the selected studies.

Study	Overall Risk of Bias	Study	Overall Risk of Bias
Adam and Knuth [40]	🟢	Reimer-Taschenbrecker et al. [36]	🟢
Antonogeorgos et al. [28]	🟢	Rennie et al. [23]	🟢
Antoñón et al. [29]	🟢	Renzi-Lomholt et al. [30]	🟢
Aratani et al. [41]	🟢	Rocco et al. [32]	🟢
Aris et al. [42]	🟢	Rodrigues et al. [33]	🟢
Aryee et al. [43]	🟢	Rogerson et al. [58]	🟢
Baek et al. [44]	🟢	Ryan et al. [59]	🟢
Caffrey-Osvald et al. [31]	🟢	Schreiber et al. [24]	🟢
Choragudi et al. [37]	🟢	Shanahan et al. [60]	🟢
Commodore et al. [45]	🟡	Sharma et al. [61]	🟢
Correa-Agudelo et al. [46]	🟢	Siegfried et al. [35]	🟢
Faison et al. [47]	🟢	Telzak et al. [62]	🟢
Grant et al. [48]	🟢	Titus et al. [63]	🟢
Grunwell et al. [49]	🟢	Tyris et al. [64]	🟢
Hauptman et al. [50]	🟢	Tyris et al. [65]	🟢
Huang et al. [51]	🟢	Wesley et al. [66]	🟢
Joy et al. [25]	🟢	Wey et al. [26]	🟢
Jung et al. [52]	🟢	Yang-Huang et al. [34]	🟢
Khan et al. [53]	🟢		
Kim et al. [27]	🟢		
Kim et al. [54]	🟢		
Le et al. [38]	🟢		
Mahdavinia et al. [39]	🟡		
Mersha et al. [55]	🟢		
Molina et al. [56]	🟢		
Pollack et al. [57]	🟢		

🟢 = low risk; 🟡 = moderate risk

## Data Availability

Data sharing is not applicable to this article.

## Supplementary Materials

The following supporting information can be downloaded at: https://www.mdpi.com/article/10.3390/epidemiologia6030056/s1, Table S1: Quality assessment of cohort studies; Table S2: Quality assessment of cross-sectional studies; Table S3: Quality assessment of ecological studies; Table S4: Quality assessment of case-crossover studies.

## Appendix A. PubMed Searching Strategy

“asthma” [Title/Abstract] OR “asthma” [Mesh] OR “allergic diseases” [Title/Abstract] OR “atopic dermatitis” [Title/Abstract] OR “Dermatitis, Atopic” [Mesh] OR “eczema” [Title/Abstract] OR “Eczema” [Mesh] OR “food allergy” [Title/Abstract] OR “Food Hypersensitivity” [Mesh] OR “Rhinitis, Allergic, Seasonal” [Mesh] OR “allergic rhinitis” [Title/Abstract]) AND (“hospital*” [Title/Abstract] OR “Emergency department” [Title/Abstract] OR “morbidity” [Title/Abstract] OR “Hospitals” [Mesh]) AND (“child*” [Title/Abstract] OR “adolescent*” [Title/Abstract] OR “pediatric*” [Title/Abstract] OR “Child” [Mesh] OR “child, preschool” [Mesh] OR “Adolescent” [Mesh]) AND (“community-level information” [Title/Abstract] OR “population-level data” [Title/Abstract] OR “educational level” [Title/Abstract] OR “housing quality” [Title/Abstract] OR “environmental conditions” [Title/Abstract] OR “racism” [Title/Abstract] OR “geographic information system” [Title/Abstract] OR “residence characteristics” [Title/Abstract] OR “neighborhood*” [Title/Abstract] OR “violence” [Title/Abstract] OR “socioeconomic factor*” [Title/Abstract] OR “residence characteristics” [Mesh] OR “Geographic Information Systems” [Mesh] OR “geographic information systems*” [Title/Abstract]
