# Supplementary material for: Social Determinants of Health in Pediatric Asthma and Allergic Diseases: A Systematic Review
Source: Epidemiologia (Basel). 2025 Sep 11;6(3):56. doi: 10.3390/epidemiologia6030056 (PMC12452756; doi:10.3390/epidemiologia6030056)
Supplement: Supplementary file 1 [file epidemiologia-06-00056-s001.zip › epidemiologia-3625559-supplementary.pdf]

| **Table S1. Quality assessment of cohort studies** | | | | | | | | | | |
|---|---|---|---|---|---|---|---|---|---|---|
| Newcastle-Ottawa quality assessment scale for cohort studies | | | | | | | | | | |
| Author | Selection | | | | Comparability | Outcomes | | | TOTAL (maximum 9★) | Overall Quality Assessment (AHRQ) |
| | Representativeness of exposed cohort | Selection of non-exposed cohort | Ascertainment of exposure | Outcome not present at start of study | | Assessment of outcomes | Length of follow-up | Adequacy of follow-up | | |
| Aratani et al. [41] | ★ | ★ | ★ | ★ | ★★ | ★ | ★ | ★ | 9★ | Good |
| Aris et al. [42] | ★ | ★ | ★ | ★ | ★★ | - | ★ | ★ | 8★ | Good |
| Baek et al. [44] | ★ | ★ | ★ | ★ | ★★ | ★ | ★ | ★ | 9★ | Good |
| Caffrey-Osvald et al. [31] | ★ | ★ | ★ | ★ | ★★ | ★ | ★ | ★ | 9★ | Good |
| Commodore et al. [45] | ★ | ★ | - | - | ★★ | - | - | - | 4★ | Fair |
| Correa-Agudelo et al. [46] | ★ | ★ | ★ | ★ | ★★ | ★ | ★ | ★ | 9★ | Good |
| Faison et al. [47] | ★ | ★ | ★ | ★ | ★★ | ★ | ★ | ★ | 9★ | Good |
| Grant et al. [48] | ★ | ★ | ★ | ★ | ★★ | ★ | ★ | ★ | 9★ | Good |
| Grunwell et al. [49] | ★ | ★ | ★ | ★ | ★★ | ★ | ★ | ★ | 9★ | Good |
| Hauptman et al. [50] | ★ | ★ | ★ | ★ | ★★ | - | ★ | ★ | 8★ | Good |

| | | | | | | | | | | |
|---|---|---|---|---|---|---|---|---|---|---|
| Jung et al. [52] | ★ | ★ | ★ | ★ | ★★ | ★ | ★ | ★ | 9★ | Good |
| Kim et al. [54] | ★ | ★ | ★ | ★ | ★★ | ★ | ★ | ★ | 9★ | Good |
| Mahdavinia et al. [39] | ★ | ★ | ★ | - | ★★ | - | - | - | 5★ | Fair |
| Mersha et al. [55] | ★ | ★ | ★ | ★ | ★★ | ★ | ★ | ★ | 9★ | Good |
| Pollack et al. [57] | ★ | ★ | ★ | ★ | ★★ | - | ★ | ★ | 8★ | Good |
| Renzi-Lomholt et al. [30] | ★ | ★ | ★ | ★ | ★★ | ★ | ★ | ★ | 9★ | Good |
| Rogerson et al. [58] | ★ | ★ | ★ | ★ | ★★ | ★ | ★ | ★ | 9★ | Good |
| Ryan et al. [59] | ★ | ★ | ★ | ★ | ★★ | - | ★ | ★ | 8★ | Good |
| Siegfried et al. [35] | ★ | ★ | ★ | ★ | ★★ | ★ | ★ | ★ | 9★ | Good |
| Titus et al. [63] | ★ | ★ | ★ | ★ | ★★ | ★ | ★ | ★ | 9★ | Good |
| Yang-Huang et al. [34] | ★ | ★ | - | ★ | ★★ | - | ★ | ★ | 7★ | Good |

**Table S2. Quality assessment of cross-sectional studies**

Axis tool (Appraisal tool for Cross-Sectional Studies)

| Author | Introduction | Methods | | | | | | | | | Results | | | | | Discussion | | Other | | Total Yes | Quality |
|---|---|---|---|---|---|---|---|---|---|---|---|---|---|---|---|---|---|---|---|---|---|
| | 1. | 2. | 3. | 4. | 5. | 6. | 7. | 8. | 9. | 10. | 11. | 12. | 13. | 14. | 15. | 16. | 17. | 18. | 19. | 20. | | |
| Antonogeorgos et al. [28] | Y | Y | Y | Y | Y | Y | N | Y | Y | Y | Y | Y | U | N | Y | Y | Y | Y | Y | Y | 17/20 | Good |

| | | | | | | | | | | | | | | | | | | | | | |
|---|---|---|---|---|---|---|---|---|---|---|---|---|---|---|---|---|---|---|---|---|---|
| Antoñón et al. et al. [29] | Y | Y | Y | Y | Y | Y | N | Y | Y | Y | Y | Y | U | N | Y | Y | Y | Y | Y | Y | 17/20 | Good |
| Aryee et al. [43] | Y | Y | N | Y | Y | Y | N | Y | Y | Y | Y | Y | U | N | Y | Y | Y | Y | Y | Y | 16/20 | Good |
| Choragudi et al. [37] | Y | Y | N | Y | Y | Y | N | Y | Y | Y | Y | Y | U | N | Y | Y | Y | Y | Y | Y | 16/20 | Good |
| Joy et al. [25] | Y | Y | Y | Y | Y | Y | N | Y | Y | Y | Y | Y | U | N | Y | Y | Y | Y | Y | Y | 17/20 | Good |
| Khan et al. [53] | Y | Y | N | Y | Y | U | N | Y | Y | Y | Y | Y | U | N | Y | Y | Y | Y | Y | Y | 15/20 | Good |
| Kim et al. [27] | Y | Y | N | Y | Y | Y | N | Y | Y | Y | Y | Y | U | N | Y | Y | Y | Y | Y | Y | 16/20 | Good |
| Le et al. [38] | Y | Y | N | Y | Y | Y | N | Y | Y | Y | Y | Y | U | N | Y | Y | Y | Y | Y | Y | 16/20 | Good |
| Molina et al. [56] | Y | Y | N | Y | Y | Y | N | Y | Y | Y | Y | Y | U | N | Y | Y | Y | Y | Y | Y | 16/20 | Good |
| Reimer-Taschenbrecker et al. [36] | Y | Y | N | Y | Y | U | N | Y | Y | Y | Y | Y | U | N | Y | Y | Y | Y | Y | Y | 15/20 | Good |
| Rennie et al. [23] | Y | Y | N | Y | Y | Y | N | Y | Y | Y | Y | Y | U | N | Y | Y | Y | Y | Y | Y | 16/20 | Good |
| Rocco et al. [32] | Y | Y | N | Y | Y | Y | N | Y | Y | Y | Y | Y | U | N | Y | Y | Y | Y | Y | Y | 16/20 | Good |
| Schreiber et al. [24] | Y | Y | N | Y | Y | U | N | Y | Y | Y | Y | Y | U | N | Y | Y | Y | Y | Y | Y | 15/20 | Good |
| Shanahan et al. [60] | Y | Y | N | Y | Y | U | N | Y | Y | Y | Y | Y | U | N | Y | Y | Y | Y | Y | Y | 15/20 | Good |
| Telzak et al. [62] | Y | Y | N | Y | Y | U | N | Y | Y | Y | Y | Y | U | N | Y | Y | Y | Y | Y | Y | 15/20 | Good |
| Tyris et al. [64] | Y | Y | N | Y | Y | Y | N | Y | Y | Y | Y | Y | U | N | Y | Y | Y | Y | Y | Y | 16/20 | Good |
| Tyris et al. [65] | Y | Y | N | Y | Y | Y | N | Y | Y | Y | Y | Y | U | N | Y | Y | Y | Y | Y | Y | 16/20 | Good |
| Wey et al. [26] | Y | Y | Y | Y | Y | Y | N | Y | Y | Y | Y | Y | U | N | Y | Y | Y | Y | Y | Y | 17/20 | Good |

Y: Yes, N: No, U: Unclear

**Table S3. Quality assessment of ecological studies**

| Author | Category | Criterion | Score (out of 1) | Total score | Quality |
|---|---|---|---|---|---|

| | | | | | |
|---|---|---|---|---|---|
| Adam and Knuth [40] | Study Design (maximum 12 points) | Design | 0 | 15/21 | Good |
| | | Sample size | 1 | | |
| | | | | | |
| | | Unbiased inclusion of units | 1 | | |
| | | Level of data aggregation | 1 | | |
| | | Level of inference | 1 | | |
| | | Prespecification of ecologic units | 1 | | |
| | | Outcomes of interest included | 1 | | |
| | | Source of data | 1 | | |
| | Statistical methodology (maximum 6 points) | Analytic methodology | 2 | | |
| | | Validity of regression | 1 | | |
| | | Use of covariates | 1 | | |
| | | Proper adjustment for covariates | 1 | | |
| | Quality of reporting (maximum 3 points) | Statement of study design | 1 | | |
| | | Justification of study design | 1 | | |
| | | Discussion of cross-level bias and limitations | | | |
| | | | **Score (out of 1)** | **Total score** | **Quality** |
| Rodrigues et al. [33] | Study Design | Design | 1 | 19/21 | Good |
| | | Sample size | 2 | | |

| | | | | | |
|---|---|---|---|---|---|
| | | Unbiased inclusion of units | 2 | | |
| | | Level of data aggregation | 1 | | |
| | | Level of inference | 1 | | |
| | | Prespecification of ecologic units | 1 | | |
| | | Outcomes of interest included | 1 | | |
| | | Source of data | 2 | | |
| | Statistical Methodology | Analytic methodology | 2 | | |
| | | Validity of regression | 1 | | |
| | | Use of covariates | 1 | | |
| | | Proper adjustment for covariates | 1 | | |
| | Quality of Reporting | Statement of study design | 1 | | |
| | | Justification of study design | 1 | | |
| | | Discussion of cross-level bias and limitations | 1 | | |
| | | | **Score (out of 1)** | **Total score** | **Quality** |
| Wesley et al. [66] | Study Design | Design | 1 | 19/21 | Good |
| | | Sample size | 2 | | |
| | | Unbiased inclusion of units | 2 | | |
| | | Level of data aggregation | 1 | | |
| | | Level of inference | 1 | | |

| | | | |
|---|---|---|---|
| | | Prespecification of ecologic units | 1 |
| | | Outcomes of interest included | 1 |
| | | Source of data | 2 |
| | Statistical Methodology | Analytic methodology | 2 |
| | | Validity of regression | 1 |
| | | Use of covariates | 1 |
| | | Proper adjustment for covariates | 1 |
| | Quality of Reporting | Statement of study design | 1 |
| | | Justification of study design | 1 |
| | | Discussion of cross-level bias and limitations | 1 |

**Table S4. Quality assessment of case-crossover studies**

| **Author** | | **Criterion** | **Assessment & Justification** | **Total score (Low)** | **Risk of bias** |
|---|---|---|---|---|---|
| Huang et al. [51] | 1 | Appropriate case-crossover design | Low | 7/9 | Low |
| | 2 | Randomized order of treatment periods | High | | |
| | 3 | Carry-over effects addressed | Low | | |
| | 4 | Unbiased selection of case/control periods | Low | | |

| | | | | | |
|---|---|---|---|---|---|
| | 5 | Allocation concealment | High | | |
| | 6 | Blinding of outcome assessment | Low | | |
| | 7 | Incomplete outcome data handled properly | Low | | |
| | 8 | Selective outcome reporting avoided | Low | | |
| | 9 | Other bias (e.g., modeling issues, confounding) | Low | | |
| | | **Criterion** | **Assessment & Justification** | **Total score (Low)** | **Risk of bias** |
| Sharma et al. [61] | 1 | Appropriate case-crossover design | Low | 7/9 | Low |
| | 2 | Randomized order of treatment periods | High | | |
| | 3 | Carry-over effects addressed | Low | | |
| | 4 | Unbiased selection of case/control periods | Low | | |
| | 5 | Allocation concealment | High | | |
| | 6 | Blinding of outcome assessment | Low | | |
| | 7 | Incomplete outcome data handled properly | Low | | |
| | 8 | Selective outcome reporting avoided | Low | | |

| | | |
|---|---|---|
| 9 | Other bias (e.g., modeling issues, confounding) | Low |
